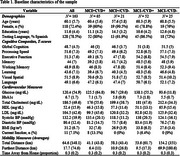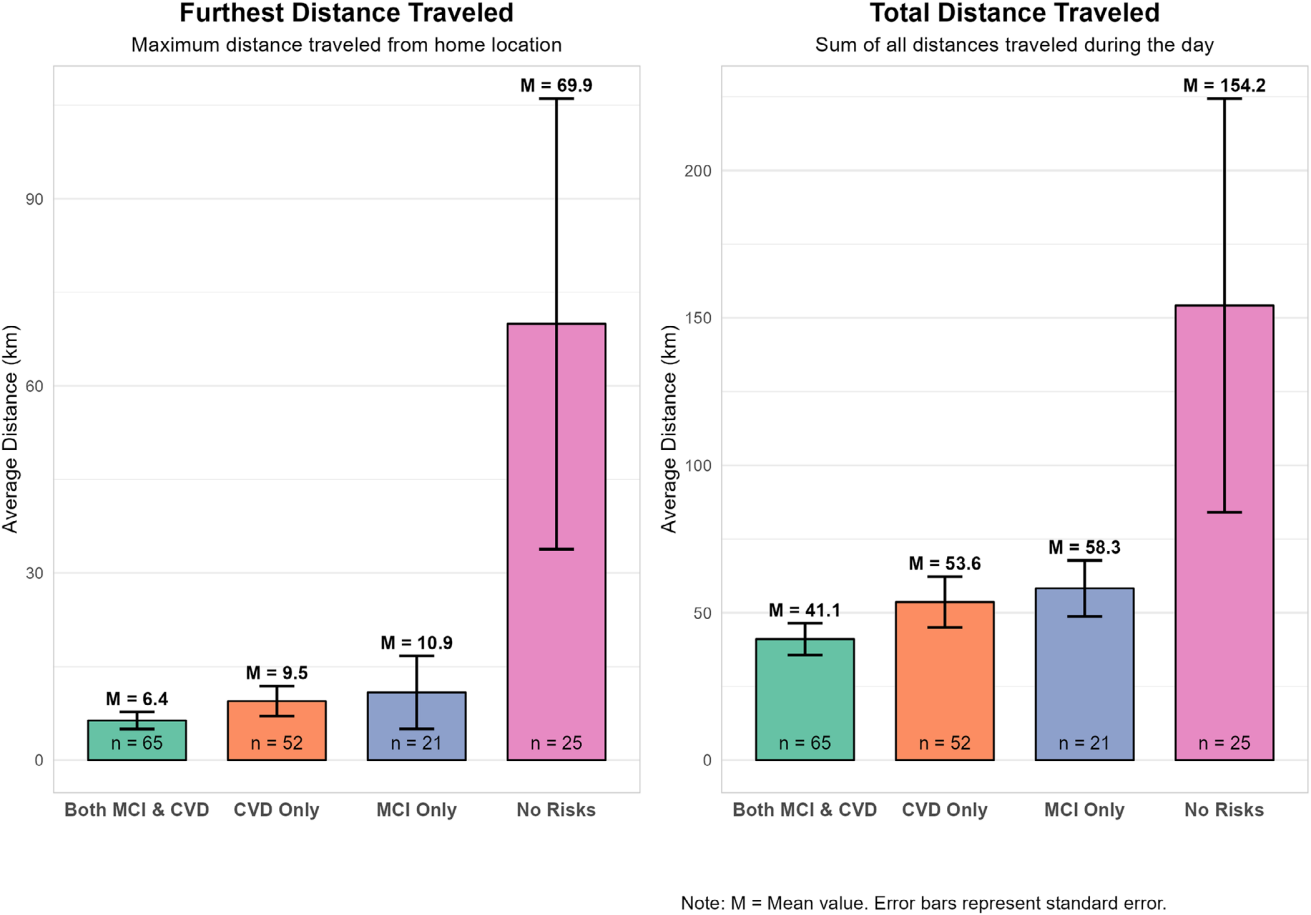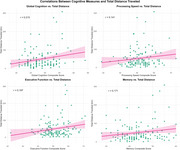# Novel Patterns of GPS‐Derived Mobility in Latino Adults with Varying ADRD Risk

**DOI:** 10.1002/alz70861_108477

**Published:** 2025-12-23

**Authors:** Andrea Mendez Colmenares, Emma Churchill, Tess Filip, Lizbeth V Murillo, Linda C Gallo, Alexander P Demos, Erin E. Sundermann, Douglas R. Galasko, Luis Betancourt, Kassandra Portillo, Rebecca Daly, Raeanne C Moore, Maria J. Marquine

**Affiliations:** ^1^ Duke University School of Medicine, Durham, NC USA; ^2^ University of Central Florida, Orlando, FL USA; ^3^ University of Iowa, Iowa City, IA USA; ^4^ San Diego State University, San Diego, CA USA; ^5^ University of Illinois at Chicago, Chicago, IL USA; ^6^ University of California, San Diego, La Jolla, CA USA

## Abstract

**Background:**

Physical mobility reflects how we navigate our environment and can reveal subtle changes in cognitive function as we age. Latino adults face disproportionate risk for Alzheimer's disease and related dementias (ADRD), yet mobility patterns in this population remain largely unexplored. Previous studies suggest connections between life‐space mobility and cognitive health, but most rely on self‐reported measures or include small samples. Smartphone GPS technology offers objective measurement of real‐world mobility patterns, yet there are very limited studies examining mobility patterns across varying cognitive and cardiovascular risk profiles, especially in diverse populations.

**Method:**

We recruited 163 Latino adults aged 50‐70 (mean age=60.1 years, 72% female) who completed comprehensive neuropsychological and medical evaluations during an in‐person baseline visit. Using established criteria, we classified participants into four ADRD risk groups based on the presence or absence of mild cognitive impairment (MCI) and cardiovascular disease (CVD) risk: MCI+/CVD+, MCI‐/CVD+, MCI+/CVD‐, and MCI‐/CVD‐. Participants installed a GPS tracking application on their personal smartphones. The application passively collected high‐frequency GPS data every 5 minutes, 24/7, with individual participants providing data for varying durations (range: 7‐30 days). We analyzed average mobility metrics and their cross‐sectional associations with cognitive performance and ADRD risk profiles.

**Result:**

Table 1 shows descriptive characteristics of the sample. Participants with both cognitive and cardiovascular risk factors showed poorer cognitive performance, especially in memory (39.2 vs 48.0 in no‐risk group). We examined GPS mobility patterns across four risk groups (MCI+/CVD+, MCI+/CVD‐, MCI‐/CVD+, MCI‐/CVD‐). The no‐risk, MCI‐/CVD‐ group traveled significantly greater distances (furthest: 69.9 km; total: 154.2 km) compared to risk‐positive groups (furthest: 6.4‐10.9 km; total: 41.1‐58.3 km), Figure 1. Total daily distance traveled was associated with better cognitive performance across several domains, with the strongest associations for global cognition (r=0.215), executive function (r=0.197), and memory (r=0.171), Figure 2.

**Conclusion:**

GPS‐derived mobility patterns reveal distinctive signatures across ADRD risk profiles in Latino adults. These digital biomarkers enable passive, continuous assessment of real‐world mobility as a functional "social marker" of how individuals navigate their environment. Smartphone‐based geospatial monitoring offers a scalable approach for detecting subtle behavioral changes that may precede clinical symptoms, potentially enhancing early ADRD detection.